# Mechanism of Buzhong Yiqi Decoction Modulates the Wnt5a/β-catenin Signaling Pathway to Ameliorate Thyroid Inflammatory Damage in Mice with Autoimmune Thyroiditis

**DOI:** 10.2174/0118715303382098250831204645

**Published:** 2026-03-09

**Authors:** Hao Gao, Ziyu Liu, Zhuo Zhao, Jiayun Li, Zhimin Wang, Huimin Cao, Yiran Chen, Si Chen, Zhe Jin, Xiao Yang

**Affiliations:** 1 School of Graduate Studies at Liaoning University of Traditional Chinese Medicine, Shenyang 110847, China;; 2 Department of Traditional Chinese Medicine Pharmacy, Liaoning Academy of Traditional Chinese Medicine, Second Affiliated Hospital, Liaoning University of Traditional Chinese Medicine, Shenyang 110034, China;; 3 Department of Endocrinol, Affiliated Hospital, Liaoning University of Traditional Chinese Medicine, Shenyang 110034, China;; 4 Department of Chinese Medicine Innovation Engineering Technology Center, Liaoning University of Traditional Chinese Medicine, Shenyang 110847, China;; 5 Department of Acupuncture and Moxibustion, College of Acupuncture and Tuina, Liaoning University of Chinese Medicine, Shenyang 110847, China;; 6 Department of Endocrinology, Second Affiliated Hospital, Liaoning University of Traditional Chinese Medicine, Shenyang 110034, China

**Keywords:** Buzhong Yiqi decoction, autoimmune thyroiditis, Wnt5a/β-catenin signaling pathway, inflammation, serum antibodies, thyroid pathology

## Abstract

**Introduction:**

The Objective is to explore the mechanism by which Buzhong Yiqi decoction improves inflammatory damage in autoimmune thyroiditis (AIT) mice based on the Wnt5a/β-catenin signaling pathway.

**Methods:**

Sixty 8-week-old NOD.H-2h4 mice of SPF grade were selected and randomly divided into six groups: control group, model group, Buzhong Yiqi decoction low, middle, and high-dose groups (4.78, 9.56, 19.12 g·kg^-1^), and Western medicine group (selenium yeast tablets, 3.033×10^-5^ g·kg^-1^), with ten mice in each group. All groups of AIT model mice were allowed to drink a 0.05% sodium iodide solution freely for 8 weeks to establish the AIT model, while the control group drank distilled water freely. ELISA, HE staining, Real-time PCR, and Western Blot were used to assess serum antibodies, thyroid pathology, and expression levels of Wnt5a, β-catenin, PPAR, GSK-3β, IL-1β, and IL-6.

**Results:**

The model group showed increased TgAb and TPOAb levels (*P*<0.01), lymphocyte infiltration, and altered expression of Wnt5a, β-catenin, PPAR, GSK-3β, IL-1β, and IL-6 (*P*<0.01). Buzhong Yiqi decoction groups and Western medicine groups significantly reduced these effects, indicating improved thyroid function and inflammation reduction.

**Discussion:**

Buzhong Yiqi decoction modulates the Wnt5a/β-catenin pathway, suggesting a potential therapeutic approach for AIT by reducing inflammation and restoring thyroid function.

**Conclusion:**

Buzhong Yiqi decoction can effectively improve AIT inflammatory damage, and the modulation of the Wnt/β-catenin signaling pathway may be one of its intervention mechanisms.

## 1 INTRODUCTION

Autoimmune thyroiditis (AIT) is a common organ-specific autoimmune disease caused by the loss of immune tolerance and the immune system’s erroneous attack on thyroid tissue [1], characterized by elevated levels of thyroglobulin antibody (TgAb) and thyroid peroxidase antibody (TPOAb), as well as histological features including lymphoplasmacytic infiltration, fibrosis, lymphoid follicle formation, and parenchymal atrophy. Hashimoto's thyroiditis (HT) is the most common clinical manifestation [2]. Epidemiological studies show that the positive rates of TPOAb and TgAb in the Chinese adult population are 11.5% and 12.6%, respectively, and the prevalence increases with age [3]. As the disease progresses, patients with AIT may develop hypothyroidism and even multisystem complications such as myxedema, hypothyroid cardiomyopathy, and metabolic disorders. Some scholars also believe that AIT is a risk factor for thyroid cancer [4, 5]. This condition seriously affects patients’ quality of life and increases the consumption of social medical resources, attracting wide attention in recent years.

At present, symptomatic treatment with thyroid hormone is a recognized therapy for AIT, but there is still a lack of specific drugs targeting its pathogenesis. Recent studies have shown that traditional Chinese medicine (TCM) exerts unique advantages in improving thyroid tissue damage and clinical symptoms from the perspective of pathogenesis [6, 7]. Based on years of clinical experience in the diagnosis and treatment of this disease, the research group concluded that the pathogenesis of AIT centers on spleen deficiency, and Buzhong Yiqi decoction, which is effective at tonifying qi and strengthening the spleen, is a beneficial prescription [8]. In addition, previous studies have found that Buzhong Yiqi decoction can intervene in the AIT inflammatory injury process through various mechanisms, such as regulating the immune imbalance of T helper 17 cells (Th17) / regulatory T cells (Treg) [9-11], with significant therapeutic effects. Therefore, further exploration of the mechanisms and related targets of Buzhong Yiqi decoction is of profound significance. The Wnt signaling pathway is an important link in many physiological and pathological processes, such as inflammation and cancer [12, 13], and has been considered a potential therapeutic target for rheumatoid arthritis and other autoimmune diseases [14]. Wu *et al.* found that in the pathogenesis of HT, inflammatory cytokines such as interferon gamma (IFN-γ) can activate glycogen synthase kinase-3 beta (GSK-3β) signal transduction molecules, increase the degradation of β-catenin, and inhibit the Wnt signaling pathway [15]. Fu *et al.* found in lung adenocarcinoma A549 cells that Buzhong Yiqi decoction could play a role by activating GSK-3β-mediated Wnt/β-catenin signaling pathway [16]. However, whether Buzhong Yiqi decoction can intervene in the development of AIT by activating the Wnt/β-catenin signaling pathway has not been confirmed. Therefore, on the basis of previous studies, this study took inflammatory injury as the entry point to explore the mechanism of Buzhong Yiqi decoction inhibiting AIT inflammation by activating the Wnt/β-catenin signaling pathway, aiming to provide a theoretical and experimental basis for the prevention and treatment of AIT with Buzhong Yiqi decoction.

## 2 MATERIALS AND METHODS

### 2.1 Experimental Animals

Sixty 8-week-old SPF NOD.H-2h4 mice, half males and half females, were randomly divided into six groups: blank group, model group, low-, middle-, and high-dose Buzhong Yiqi decoction groups, and SYT group, with 10 mice in each group (5 males and 5 females per group). The AIT model was established by allowing the mice to drink a 0.05% sodium iodide solution for 8 weeks. After model establishment, each treatment group received the corresponding doses of medication by gavage for 8 weeks, while the blank and model groups were given distilled water for the same duration. The mice, weighing (20 ± 2) g, were purchased from Jackson Laboratory (strain No. 004447, animal certificate No. 2211A01937) and bred in the SPF laboratory of the Laboratory Animal Center of Liaoning University of Traditional Chinese Medicine. The indoor temperature was maintained at 20–25 °C, relative humidity at 50% ± 20%, with alternating light and dark cycles. The animal experiment in this study was approved by the Ethics Committee of Liaoning University of Traditional Chinese Medicine (ethical review No. 21000042021128). We strictly adhered to the national guidelines for the care and use of laboratory animals.

### 2.2 Experimental Drugs

Ingredients: Radix astragali (18 g), Atractylodes (9 g), Glycyrrhizae Radix Praeparata (9 g), Panax ginseng (6 g), Citrus reticulata peel (6 g), cohosh (6 g), Radix bupleuri (6 g), Angelica sinensis (3 g). These herbal pieces were produced by Anhui Puren TCM Decoction Pieces Co., LTD. They were purchased from the Affiliated Hospital of Liaoning University of Traditional Chinese Medicine and identified by Professor Yang Xiao of the Second Affiliated Hospital of Liaoning University of Traditional Chinese Medicine. All meet the requirements of the 2020 edition of the Pharmacopoeia of the People's Republic of China. The decoction procedure was as follows: the required herbal pieces were immersed in eight volumes of distilled water (about 480 mL) for 1 hour, then boiled over medium heat, and an appropriate amount of distilled water was added; the mixture was gently decocted for 30 minutes, filtered; the filtrates were combined and concentrated to 1.0 g/mL. Positive control drug: selenium yeast tablets (Mudanjiang Lingtai Pharmaceutical Co., LTD.), specification 50 μg/tablet, Chinese medicine approval number H10940161. The tablets were ground before use, and a suspension with a concentration of 1.625 mg/L was prepared and stored in the refrigerator at 4°C for use.

### 2.3 Main Reagents and Instruments

Wnt5a Polyclonal Antibody, β-catenin Polyclonal Antibody, PPARA Monoclonal Antibody, GSK-3β Polyclonal Antibody, IL-1β Polyclonal Antibody, IL-6 Polyclonal Antibody, and GAPDH Monoclonal Antibody were purchased from Wuhan Sanying Biotechnology Co., Ltd., with batch numbers 55184-1-AP; 51067-2-AP; 66826-1-Ig; 14850-1-AP; 16806-1-AP; 21865-1-AP; and 60004-1-Ig, respectively. The HiFiScript gDNA Removal cDNA Synthesis Kit and MagicSYBR Mixture PCR Amplification Kit were obtained from Beijing Kangwei Century Biotechnology Co., Ltd., with batch numbers 19223 and 17623, respectively. PCR primers were designed and synthesized by Dalian Bao Biological Co., Ltd. The SpectraMax i3 multifunctional full-wavelength microplate reader was purchased from Meigu Molecular Instruments Co., Ltd. The THZ-82 water bath oscillator was obtained from Changzhou Zhiborui Company. WIX-miniPRO4 vertical electrophoresis cell and WIX-miniBLOT transfer cell were supplied by Beijing Vickers Company. The Tanon-5200 development instrument was from Shanghai Tanon Company. The computer-automated tissue dehydrator was purchased from Changzhou Hao Silin Medical Instrument Co., Ltd. The YD-6L biological tissue freezing table, YD-A biological tissue spreading machine, and YD-B biological tissue baking machine were obtained from Jinhua Ydi Medical Equipment Co., Ltd. Additional equipment included a microtome (Leica, Germany), biomicroscope (Nikon Corporation, Japan), ST16R ultracentrifuge, ABI 7500 real-time fluorescence quantitative PCR instrument, and ultra-micro ultraviolet spectrophotometer (Thermo Fish).

### 2.4 AIT Model Establishment and Grouping

The mice in the control group were fed distilled water, and the mice in the other groups were treated according to international modeling standards [17]. Based on our previous research, a 0.05% sodium iodide solution was provided ad libitum for drinking for 8 weeks. Microscopic examination revealed a large amount of lymphocyte infiltration in thyroid tissue, and increased titres of serum autoimmune antibodies were observed, indicating successful establishment of the model [18]. After the model was successfully established, the mice in the low-, middle-, and high-dose Buzhong Yiqi decoction groups were treated with Buzhong Yiqi decoction at doses of 4.78 g·kg ^-1^, 9.56 g·kg ^-1^, and 19.12 g·kg ^-1^, respectively, with the middle dose equivalent to the clinical dose used in humans. In the SYT group was given by gavage at a dose of 3.033 × 10^-5^ g·kg ^-1^. The mice in the other groups were treated with the same volume of distilled water once a day for 8 weeks.

### 2.5 HE was used to Detect The Pathological Status of Thyroid Tissue

The thyroid tissues of mice in each group were fixed with 4% paraformaldehyde, cleared in xylene, and dehydrated through a graded ethanol series. Then, the tissues were stained with hematoxylin for 5 min, differentiated until the nuclei turned blue, dehydrated again through a graded ethanol series, stained with eosin for 5 min, and finally mounted with neutral gum. The degree of inflammatory cell infiltration in thyroid tissues of each group was evaluated under a light microscope.

### 2.6 The Levels of TGAb and TPOAb in Serum were Detected by ELISA

The mice were anesthetized with 1% sodium pentobarbital (50 mg/kg). After the blood samples stood for 2 hours at room temperature, the serum was separated by centrifugation at 3000 rpm for 10 minutes, with a centrifugation radius of 2.5 cm for all subsequent centrifugation steps. The serum thyroglobulin antibody (TGAb) and thyroid peroxidase antibody (TPOAb) levels were measured strictly according to the ELISA kit instructions.

### 2.7 The mRNA Expression Levels of Wnt5a, β-catenin, PPAR, GSK-3β, IL-1β, and IL-6 in the Thyroid of NOD.H-2h4 Animals were Detected by RT-qPCR

Strictly follow the TRIzol reagent manual to extract total RNA from animal thyroid cells. Then, use a UV spectrophotometer to measure the absorbance (A) at 260 nm and 280 nm, and calculate the RNA purity based on the A260/A280 ratio as well as the RNA concentration. Next, take 2 μL of total RNA as the template and use oligo-dT as the primer for reverse transcription. The reaction volume is 20 μL, and the reaction conditions are 37 °C for 15 min followed by 85 °C for 5 s. Real-time quantitative reverse transcription PCR is performed with the following cycle conditions: 95 °C for 0.5 min for 1 cycle; 95 °C for 5 s and 60 °C for 34 s for 40 cycles; then 95 °C for 15 s, 60 °C for 1 min, and 95 °C for 15 s for 1 cycle. To eliminate variations caused by sample extraction, reverse transcription, and PCR processes, the expression of the housekeeping gene β-actin is detected simultaneously. After completing the PCR cycles, analyze the data and calculate the relative expression levels of the target gene in each group using the 2 ΔΔCT method (Table **1**).

### 2.8 Western Blot Detection of Wnt5a, β-catenin, PPAR, GSK-3β, IL-1β, and IL-6 Protein Expression in the Thyroid of AIT Animals

Take thyroid tissue and homogenize it using a glass homogenizer; then add protein lysis buffer and incubate on ice for 30 minutes to lyse the cells. Centrifuge at 12,000 rpm for 5 minutes and collect the supernatant. For protein quantification, use a UV spectrophotometer to measure absorbance at 280 nm, plot a standard curve, and calculate the corresponding protein concentration. Prepare the polyacrylamide gel (SDS-PAGE). Meanwhile, add the sample to the loading buffer and boil in water for 5 minutes to fully denature the proteins. Perform SDS-PAGE electrophoresis, then transfer the proteins from the SDS-PAGE gel to a membrane. After transfer, wash the membrane twice with TBST. Block the membrane with 5% skim milk for 1 hour; after blocking, wash the membrane three times with TBS. Add the primary antibodies for Wnt5a (1:3000), β-catenin (1:5000), PPARγ (1:3000), GSK-3β (1:3000), IL-1β (1:2000), and IL-6 (1:2000), and incubate overnight at 4°C. The next day, wash the membrane three times with TBST. Prepare the secondary antibody dilution solutions according to the instructions, using rabbit secondary antibody (1:2000) and mouse secondary antibody (1:5000), and incubate on a shaker at room temperature for 1 hour. Wash the membrane three times with TBST, then develop the membrane using ECL substrate and expose to detect bands. Capture images of the bands and quantitatively analyze the grayscale values of each protein band using appropriate software.

### 2.9 Statistical Analysis

SPSS 23.0 software was used for data statistical analysis. Multiple groups of samples met the normality test and homogeneity of variance, and one-way analysis of variance was used. Measurement data were expressed as mean ± standard deviation (𝑥 ± 𝑠), *P* < 0.05 was statistically significant.

## 3 RESULTS

### 3.1 Effect of Buzhong Yiqi Decoction on *Serum* TGAb and TPOAb Levels in AIT Mice

**Fig. (1):**
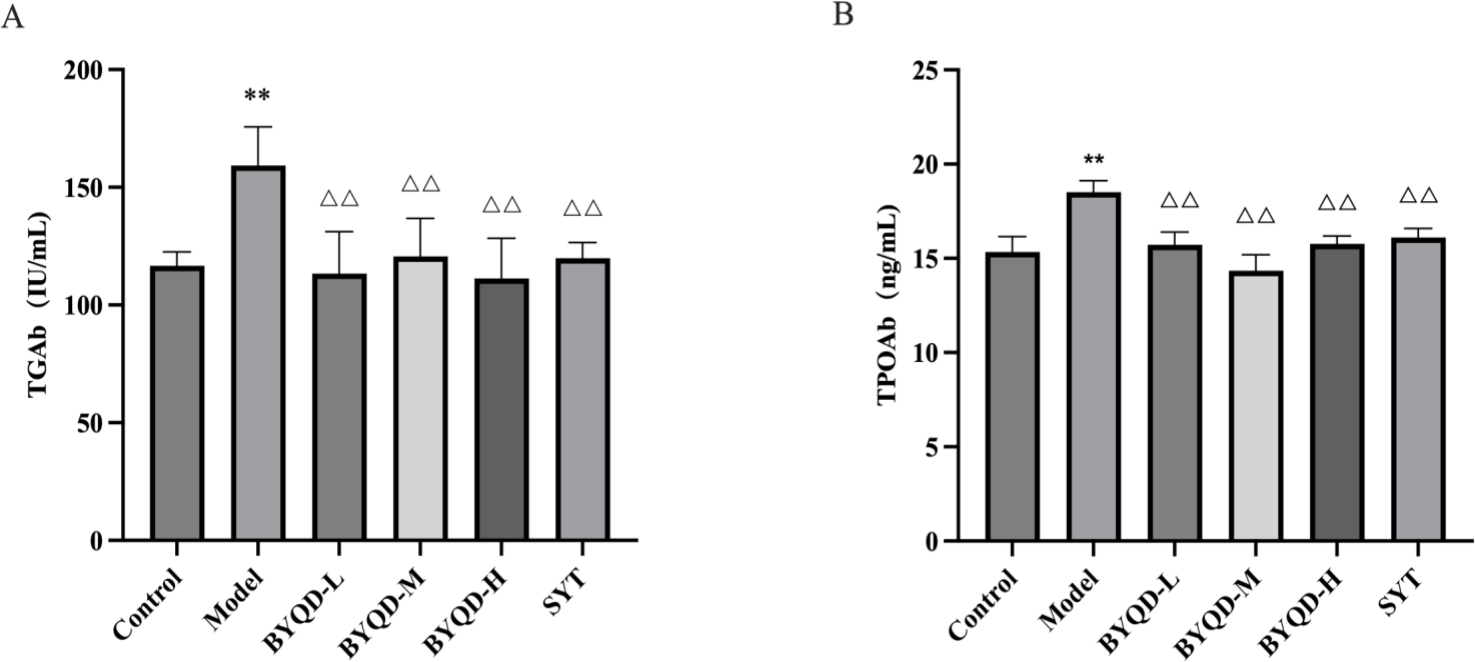
The contents of TGAb and TPOAb in serum of mice in each group. (**A**) TGAb test results. (**B**) TPOAb test results. Comparison analysis was conducted using the Student t-test and one-way ANOVA. The least significant difference method was used to assess statistical significance between the groups. The data is presented as mean ± standard deviation with a sample size of ten (n = 10). **P < 0.01 *vs*. control group; ∆∆*P* < 0.01 *vs*. model group.

Compared with the control group, the serum levels of TGAb (Fig. **1A**) and TPOAb (Fig. **1B**) in the model group were significantly increased (*P* < 0.01). Compared with the model group, the levels of serum antibodies TGAb and TPOAb in the Buzhong Yiqi decoction groups and the western medicine group were significantly decreased (*P* < 0.01).

### 3.2 Effect of Buzhong Yiqi Decoction on Thyroid Pathobiology in AIT Mice

**Fig. (2):**
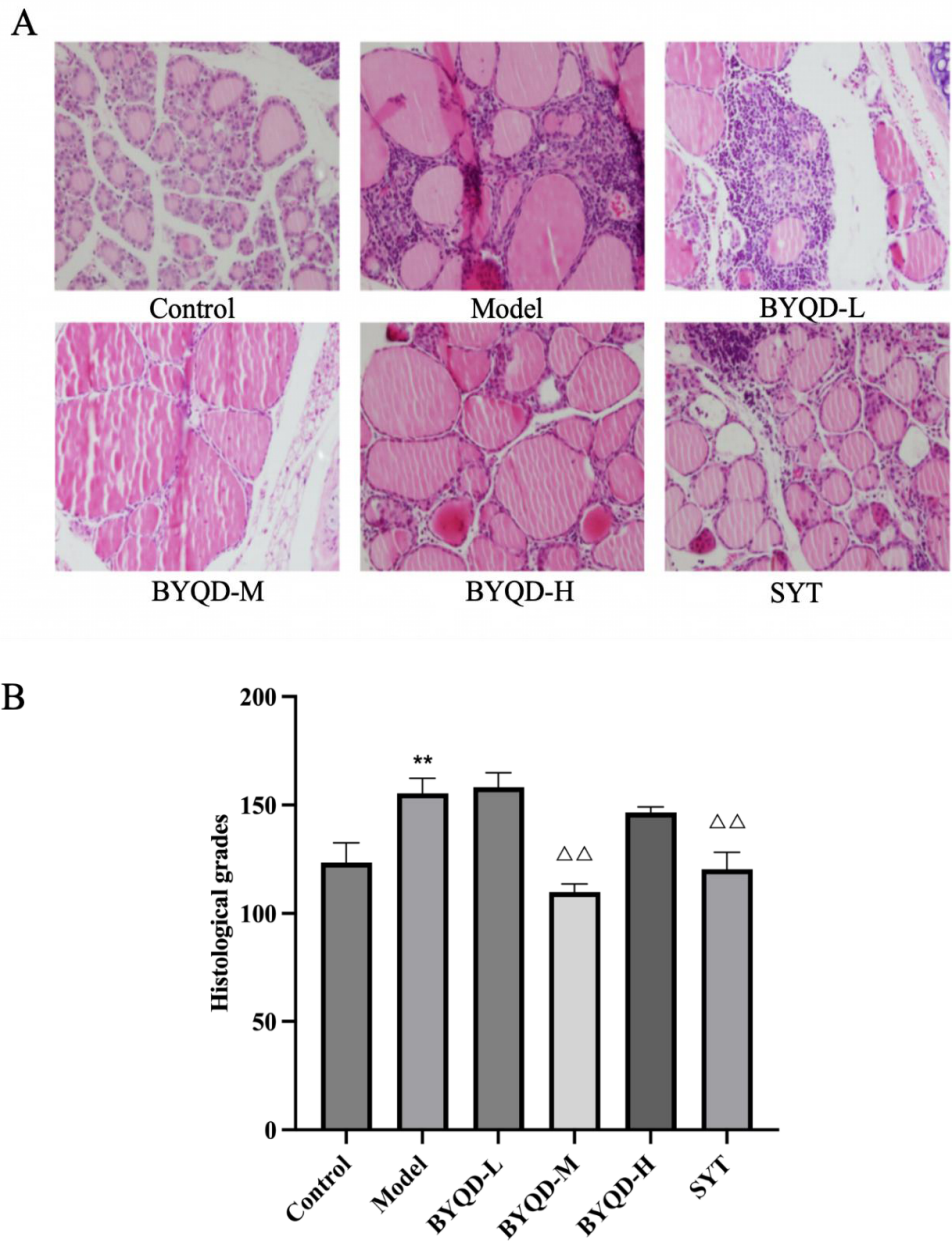
Histopathological changes of thyroid tissue in each group. (**A**) Pathological morphology of thyroid tissue. (**B**) Semi-quantitative analysis of HE images. Comparison analysis was conducted using the Student t-test and one-way ANOVA. The least significant difference method was used to assess statistical significance between the groups. The data is presented as mean ± standard deviation with a sample size of four (n = 3). **P < 0.01 *vs*. control group; ∆∆*P* < 0.01 *vs*. model group.

Mice in the control group had a complete structure and regular shape of thyroid follicles, which were round or oval in shape and similar in size, and there was no lymphocyte infiltration in the follicular space. Compared with the control group, the thyroid follicles in the model group were different in size, the follicular epithelial cells were arranged disorderly, and the morphology was different. Most of the follicular structure was destroyed and atrophic, and most of the lymphocytes were infiltrated into the follicular space. Compared with the model group, the thyroid follicular epithelial cells were arranged neatly, the follicular cavity was atrophed, the colloid content was reduced, and absorption vacuoles were seen, and the degree of lymphocyte infiltration in the space was significantly improved in the Buzhong Yiqi decoction middle-dose group (Fig. **2A**). Additionally, semi-quantitative analysis of HE images revealed significant differences in histopathological changes among the groups (Fig. **2B**).

### 3.3 The mRNA Expression of Wnt5a, β-catenin, PPAR, GSK-3β, IL-1β, and IL-6 in thyroid tissue of AIT Mice Treated with Buzhong Yiqi Decoction

Compared with the control group, Wnt5a, β-catenin, and PPAR in the model group were significantly decreased (*P* < 0.01), and GSK-3β, IL-1β, and IL-6 were significantly increased *(P* < 0.01). Compared with the model group, the levels of Wnt5a, β-catenin, and PPAR were significantly increased (*P* < 0.05, *P* < 0.01), and the levels of GSK-3β, IL-1β, and IL-6 were significantly decreased (*P* < 0.01) in each treatment group. As shown in Figs. (**3A**–**3F**).

### 3.4 The Protein Expression of Wnt5a, β-catenin, PPAR, GSK-3β, IL-1β, and IL-6 in Thyroid Tissue of AIT Mice Treated with Buzhong Yiqi Decoction

Compared with the control group, Wnt5a, β-catenin, and PPAR in the model group were significantly decreased (*P* < 0.01), and GSK-3β, IL-1β, and IL-6 were significantly increased (*P* < 0.01). Compared with the model group, the levels of Wnt5a, β-catenin, and PPAR were significantly increased (*P* < 0.01), and the levels of GSK-3β, IL-1β, and IL-6 were significantly decreased (*P* < 0.01) in each treatment group. These changes are visually represented and statistically analyzed in Figs. (**4A**-**F**), and the protein expression is depicted in Fig. (**4G**).

## 4 DISCUSSION

As one of the common autoimmune diseases, the pathogenesis of AIT remains unclear. It is believed to be closely related to environmental, genetic, and immune factors. Immunity and inflammation have been shown to play a key role throughout the entire course of AIT. Inflammation is the body's protective response against harmful stimuli such as injuries and infections.

AIT belongs to the category of “gall disease” in traditional Chinese medicine. Based on many years of clinical diagnosis and treatment experience of the research group, the concept of ancient doctors' prescriptions is condensed, and the deficiency of temper is the root of AIT. Spleen is the root of acquired natureand it not only loses its movement and defense, gradually leading to inability of body movement and prevention and control of internal and external pathogens, resulting in qi stagnation, phlegm, and blood stasis in the front of the neck, and gall disease. Therefore, based on the evidence, the treatment should be based on invigorating qi and strengthening the spleen. The ancient prescription of Buzhong Yiqi decoction came from Li Dongyuan, the four great doctors of the Jin and Yuan Dynasties. Rising Yang solid table, for the jun medicine. Compatible with ginseng, fried licorice, Atractylodes, invigorating qi, and invigorating spleen as the official medicine. Angelica nourishes blood and camp, ginseng, astragalus tonifying qi and nourishing blood; Tangerine can regulate qi and stomach, so that all the herbs can be replenished without stagnation. A small amount of cohoxime and bupleurum raises Yang to lift the depression, and assist Jun medicine to lift qi in the depression. Fried licorice and herbs are made of herbs.

As the classical Wnt pathway, the Wnt/β-catenin signaling pathway extensively regulates cell growth, migration, and differentiation, and controls the expression of downstream genes, including anti-inflammatory genes. In recent years, it has been confirmed in a variety of inflammatory injuries and autoimmune diseases. In this complex regulatory network, Wnt5a, as a major ligand, acts on a variety of classical or non-classical Wnt signaling pathways to participate in inflammatory response by binding to different receptors [19]. Among them, β-catenin is the core component of this pathway. When Wnt signaling is activated, β-catenin accumulates in the cytoplasm and combines with T cell cytokines/lymphokines to promote the transcription of downstream target genes. When Wnt signaling is inhibited, β-catenin can be continuously degraded by a “degradation complex” in the cytoplasm to maintain low levels [20-22]. GSK-3β is the most important endogenous inhibitor of Wnt/β-catenin signaling pathway. As a neuron-specific serine-threonine kinase, GSK-3β participates in the regulation of various physiological and pathological signals, such as inflammatory response [23, 24]. As a member of the nuclear receptor protein family, PPAR is composed of transcription factors involved in various processes such as glucose and lipid metabolism, and plays an important role in metabolism, growth, and development, and many physiological processes in the body [25]. Studies have shown that β-catenin, the main component of the Wnt signaling pathway, is the target of PPARγ; that is, PPARγ can activate the Wnt pathway to participate in inflammatory response by reducing the activity of GSK-3β and reducing the phosphorylation of β-catenin [26]. In recent years, it has been discovered that coumarin natural products significantly elevate the levels of anti-inflammatory cytokines such as IL-10 in colitis models by directly binding to the ATP-binding pocket of GSK-3β and inhibiting β-catenin phosphorylation and degradation [27]. Additionally, studies have revealed that the non-steroidal anti-inflammatory drug indomethacin can markedly enhance the efficiency of CD4+ T cell differentiation into the Th17 phenotype by blocking the Wnt/β-catenin signaling pathway [28]. In the organ-specific damage of Hashimoto's thyroiditis, Wnt5a has been found to activate the NLRP3 inflammasome through a non-canonical Ca2+-dependent pathway. Monoclonal antibodies targeting ROR1/2 receptors can block this abnormal activation, reducing pyroptosis of thyroid follicular epithelial cells by up to 67% in animal models [29].

The previous application of Buzhong Yiqi decoction in the clinical intervention of AIT patients has achieved significant efficacy, which can reduce the levels of TPOAb and TgAb. Animal experiments have confirmed that Buzhong Yiqi decoction can regulate Th17 cells and effectively improve the immune dysfunction state of AIT mice [30], especially the middle dose has the best effect. The reason may be that Buzhong Yiqi decoction is formulated based on the TCM theory of “tonifying the middle-jiao and replenishing qi, elevating yang qi,” primarily indicated for spleen-qi deficiency syndrome with sinking of yang qi. However, high-dose administration may deviate from the TCM principle of “discontinuing treatment upon achieving the desired effect,” potentially leading to “qi stagnation due to over-supplementation” or “disruption of qi mechanism.” High doses may elevate the risk of toxicity associated with certain herbal components or precipitate adverse reactions due to individual variability.

In this study, Buzhong Yiqi decoction was used to intervene in an AIT disease model in NOD.H-2h4 mice. The results showed that Buzhong Yiqi decoction could effectively reduce the levels of TGAb and TPOAb in AIT NOD.H-2h4 mice after 8 weeks of intervention. HE staining also showed that the pathological damage of the thyroid in each treatment group was alleviated to varying degrees compared with the model group, which could effectively reduce the degree of lymphocyte infiltration. These macroscopic indices all showed that Buzhong Yiqi decoction could improve AIT, and the middle-dose group of Buzhong Yiqi decoction had the most significant effect. Based on the network pharmacology analysis of the related targets of Buzhong Yiqi decoction, it was found that the core components of Buzhong Yiqi decoction, such as kaempferol, quercetin, naringin, and nobeletin, may act on inflammation and other processes by regulating AKT1, TNF, IL-6, IL-1β, and other related targets [31]. The mRNA and protein expression levels of IL-1β and IL-6 in thyroid tissues of AIT model mice were significantly increased, indicating that excessive intake of sodium iodide induced an inflammatory response. After treatment with different doses of Buzhong Yiqi decoction, the expression levels of Il-1β and IL-6 were significantly decreased. In addition, Buzhong Yiqi decoction could up-regulate Wnt5a, β-catenin, and PPAR, down-regulate the mRNA and protein expression of GSK-3β, alleviate the inflammatory damage of AIT, reduce the degree of lymphatic infiltration, and protect thyroid function by RT-qPCR and Western Blot.

Our team posits that Buzhong Yiqi decoction can reduce TGAb and TPOAb levels, attenuate inflammatory responses, and its therapeutic effects are quite remarkable, being most optimal at a medium dose. This effect may be linked to the activation of the Wnt/β-catenin signaling pathway. Nevertheless, the present study is still limited in its dissection of the molecular mechanism by which BYQD ameliorates AIT in mice. We have only preliminarily characterized, through *in-vivo* experiments, how the Buzhong Yiqi Decoction might prevent and treat AIT *via* this route; the precise, target-specific control of Wnt5a/β-catenin signaling in AIT-related inflammatory damage remains to be rigorously verified with activators or other tools. Whether BYQD exerts therapeutic effects on AIT by selectively modulating inflammatory injury through the Wnt5a/β-catenin pathway, and which active constituents are critically responsible, will therefore constitute the central focus of our future work.

## CONCLUSION

The present study elucidates that Buzhong Yiqi decoction significantly alleviates inflammatory injury in AIT by potentially modulating the Wnt5a/β-catenin signaling pathway. Our findings suggest that this traditional herbal formula could serve as a promising therapeutic option for AIT. The implications of our research highlight the potential of Buzhong Yiqi decoction in clinical settings, warranting further investigation into its optimization and application. Additionally, our results pave the way for future studies to explore the intricate mechanisms underlying the anti-inflammatory effects of this decoction.

## Figures and Tables

**Fig. (3):**
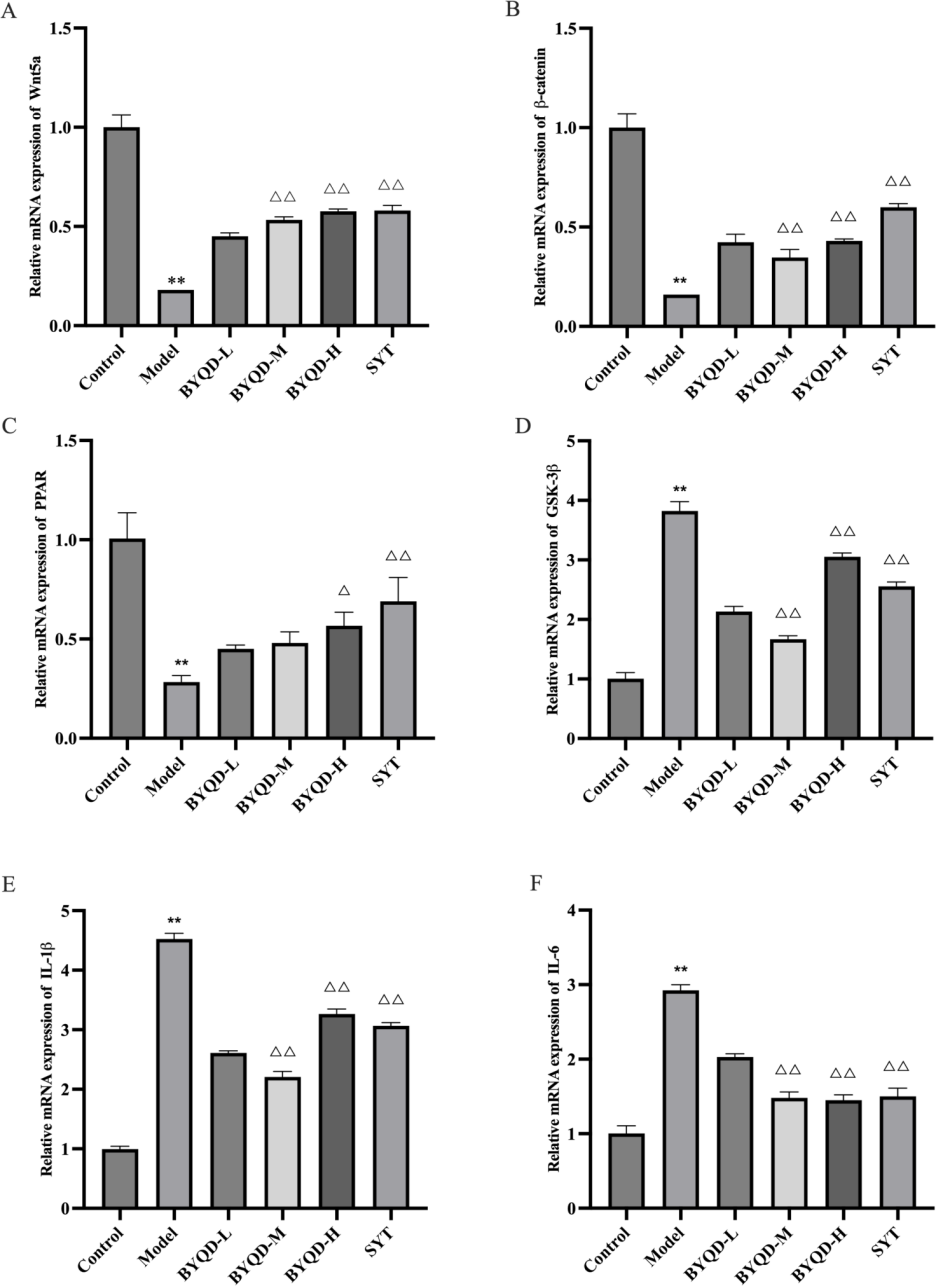
The expression of mRNA related to Wnt5a/β-catenin signal pathway in thyroid tissue of mice in each group. (**A-F**). Statistical analysis of Wnt5a, β-catenin, PPAR, GSK-3β, IL-1β, and IL-6 mRNA expression in thyroid tissue. Comparison analysis was conducted using the Student t-test and one-way ANOVA. The least significant difference method was used to assess statistical significance between the groups. The data is presented as mean ± standard deviation with a sample size of three (n = 3). **P < 0.01 *vs*. control group; ∆*P* < 0.05 and ∆∆*P* < 0.01 *vs*. model group.

**Fig. (4):**
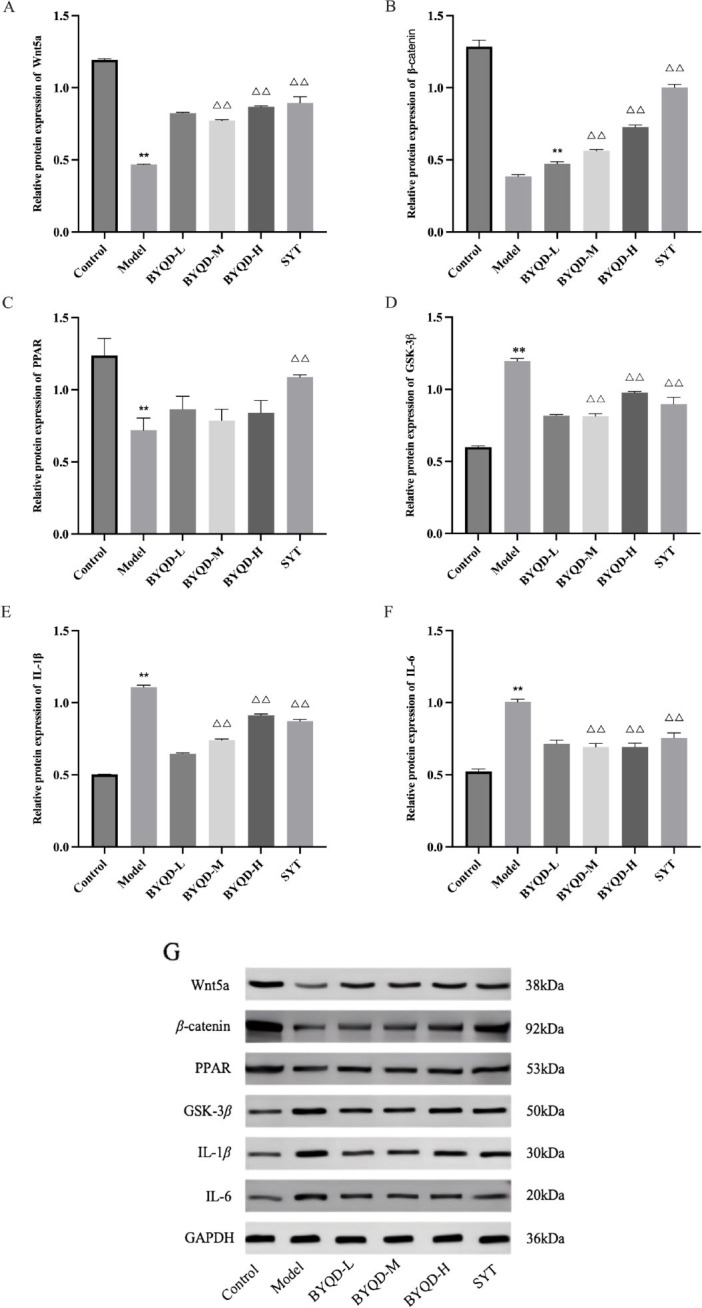
The expression of Wnt5a/β-catenin signal pathway-related protein in thyroid tissue of mice in each group. (**A-F**) Statistical analysis of related protein expression. (**G**) representative imprinting of Wnt5a, β-catenin, PPAR, GSK-3β, IL-1β and IL-6 proteins in thyroid tissues. Comparison analysis was conducted using the Student t-test and one-way ANOVA. The least significant difference method was used to assess statistical significance between the groups. The data is presented as mean ± standard deviation with a sample size of three (n = 3). **P < 0.01 *vs*. control group; ∆*P* < 0.05 and ∆∆*P* < 0.01 *vs*. model group.

**Table 1:** Primer sequence of the gene.

**Gene**	**Primer Sequence**	**bp**
Wnt5a	Forward primer:CAACTGGCAGGACTTTCTCAA	128
	Reverse primer:CATCTCCGATGCCGGAACT	-
β-catenin	Forward primer:ATGGAGCCGGACAGAAAAGC	108
	Reverse primer:CTTGCCACTCAGGGAAGGA	-
PPAR	Forward primer:AGAGCCCCATCTGTCCTCTC	153
	Reverse primer:ACTGGTAGTCTGCAAAACCAAA	-
GSK-3β	Forward primer:TGGCAGCAAGGTAACCACAG	189
	Reverse primer:CGGTTCTTAAATCGCTTGTCCTG	-
IL-1β	Forward primer:GCAACTGTTCCTGAACTCAACT	89
	Reverse primer:ATCTTTTGGGGTCCGTCAACT	-
IL-6	Forward primer:CCAAGAGGTGAGTGCTTCCC	118
	Reverse primer:CTGTTGTTCAGACTCTCTCCCT	-
β-actin	Forward primer:GGCTGTATTCCCCTCCATCG	154
	Reverse primer:CCAGTTGGTAACAATGCCATGT	-

## Data Availability

All the data and supporting information are provided within the article.

## LIST OF ABBREVIATIONS

AIT: Autoimmune Thyroiditis
TPOAb: Thyroid Peroxidase Antibody
HT: Hashimoto’s Thyroiditis
TCM: Traditional Chinese Medicine
Treg: Regulatory T Cells

## References

[1] Chen Y-K., Lin C-L., Cheng F.T-F., Sung F-C., Kao C-H. (2013). Cancer risk in patients with Hashimoto’s thyroiditis: a nationwide cohort study.. Br. J. Cancer.

[2] Giordano C., Stassi G., De Maria R., Todaro M., Richiusa P., Papoff G., Ruberti G., Bagnasco M., Testi R., Galluzzo A. (1997). Potential involvement of Fas and its ligand in the pathogenesis of Hashimoto’s thyroiditis.. Science.

[3] Shan Z., Chen L., Lian X., Liu C., Shi B., Shi L., Tong N., Wang S., Weng J., Zhao J., Teng X., Yu X., Lai Y., Wang W., Li C., Mao J., Li Y., Fan C., Teng W. (2016). Iodine status and prevalence of thyroid disorders after introduction of mandatory universal salt iodization for 16 years in China: A cross-sectional study in 10 Cities.. Thyroid.

[4] Li Y., Zang Y., Fan T., Li Z., Li A., Lv W., Wang Q., Li Q., Li Y., Li Q., Sun Z., Teng H. (2022). Transcriptomic signatures associated with autoimmune thyroiditis in papillary thyroid carcinoma and cancer immunotherapy-induced thyroid dysfunction.. Comput. Struct. Biotechnol. J..

[5] Jonklaas J., Bianco A.C., Bauer A.J., Burman K.D., Cappola A.R., Celi F.S., Cooper D.S., Kim B.W., Peeters R.P., Rosenthal M.S., Sawka A.M., American Thyroid Association Task Force on Thyroid Hormone Replacement (2014). Guidelines for the treatment of hypothyroidism: prepared by the american thyroid association task force on thyroid hormone replacement.. Thyroid.

[6] Wu M., Du G. (2020). Clinical study of Buzhong Yiqi Decoction in treatment of Hashimoto thyroiditis.. Zhongchengyao.

[7] Liu Z., Wang Z., Song N. (2022). Study on the potential mechanism of Buzhong Yiqi Decoction in treating autoimmune thyroiditis based on miRNA sequencing technology.. Zhongguo Shiyan Fangjixue Zazhi.

[8] Wang Y., Gao T. (2008). Clinical observation of 30 cases of Hashimoto thyroiditis treated by spleen deficiency and phlegm stasis.. J. New Chin. Med..

[9] Luo Y., Liu Z., Song N. (2023). Effect of Buzhong Yiqi Decoction on TLR4/NF-κB/NLRP3 signaling pathway in mice with autoimmune thyroiditis.. Lishizhen Med. Mater. Med. Res..

[10] Zhao Z., Song N., Liu Z. (2024). Effects of Buzhong Yiqi Decoction on Th17/Treg immune imbalance and Notch1 signaling pathway in AIT mice.. Zhongguo Shiyan Fangjixue Zazhi.

[11] Yang X., Wang Z.M., Cao H. (2022). Effect of Buzhong Yiqi Decoction drug-containing serum on Th17 cell differentiation in AIT mice based on miR-155/SOCS1/STAT3 axis.. Zhonghua Zhongyiyao Xuekan.

[12] Shi J., Chi S., Xue J., Yang J., Li F., Liu X. (2016). Emerging role and therapeutic implication of Wnt signaling pathways in autoimmune diseases.. J. Immunol. Res..

[13] Liu J., Xiao Q., Xiao J., Niu C., Li Y., Zhang X., Zhou Z., Shu G., Yin G. (2022). Wnt/β-catenin signalling: function, biological mechanisms, and therapeutic opportunities.. Signal Transduct. Target. Ther..

[14] Jin Z.X., Tang F., Ma W.K. (2020). Intervention mechanism of traditional Chinese medicine through Wnt/β-catenin signaling pathway in rheumatoid arthritis. *Rheun.*. Arthr..

[15] Wu F., Mao C., Mou X., Xu C., Zheng T., Bu L., Luo X., Lu Q., Wang X. (2022). Decreased β-catenin expression contributes to IFNγ-induced chemokine secretion and lymphocyte infiltration in Hashimoto’s thyroiditis.. Endocr. Connect..

[16] Fu Q.Z., Mu Q.R., Liu C.Y. (2023). Effect of Buzhong Yiqi Decoction on the cisplatin sensitivity of lung adenocarcinoma A549 cell transplantation by Wnt/β-catenin signaling pathway.. Zhongguo Laonianxue Zazhi.

[17] Braley-Mullen H., Sharp G.C., Medling B., Tang H. (1999). Spontaneous autoimmune thyroiditis in NOD.H-2h4 mice.. J. Autoimmun..

[18] Li C., Peng S., Liu X., Han C., Wang X., Jin T., Liu S., Wang W., Xie X., He X., Zhang H., Shan L., Fan C., Shan Z., Teng W. (2017). Glycyrrhizin, a direct HMGB1 antagonist, ameliorates inflammatory infiltration in a model of autoimmune thyroiditis *via* inhibition of TLR2-HMGB1 signaling.. Thyroid.

[19] Huang Y., Xue Q., Chang J., Wang X., Miao C. (2023). Wnt5a: A promising therapeutic target for inflammation, especially rheumatoid arthritis.. Cytokine.

[20] Anthony C.C., Robbins D.J., Ahmed Y., Lee E. (2020). Nuclear regulation of Wnt/β-Catenin signaling: It’s a complex situation.. Genes (Basel).

[21] Topol L., Jiang X., Choi H., Garrett-Beal L., Carolan P.J., Yang Y. (2003). Wnt-5a inhibits the canonical Wnt pathway by promoting GSK-3–independent β-catenin degradation.. J. Cell Biol..

[22] Pai S.G., Carneiro B.A., Mota J.M., Costa R., Leite C.A., Barroso-Sousa R., Kaplan J.B., Chae Y.K., Giles F.J. (2017). Wnt/beta-catenin pathway: modulating anticancer immune response.. J. Hematol. Oncol..

[23] Zhang X.L., Hu M.N., Li Y.N., Wang Y., Rong Z., Qi L., Zhou Z.Y., Ma J. (2025). Effect of electroacupuncture on the permeability of blood-brain-barrier mediated by Wnt7a/β-catenin/GSK-3β pathway in mice with Parkinson’s disease.. Zhen Ci Yan Jiu.

[24] Wang J.Q., Dong Y., Feng Z.M., Fan M.L., Yang J.Y., Hu J.N., Cai E.B., Zhu H.Y., Li W., Wang Z. (2023). Ginsenoside Re attenuates cisplatin-induced intestinal toxicity *via* suppressing GSK-3β-dependent Wnt/β-catenin signaling pathway *in vivo* and *in vitro.*. Am. J. Chin. Med..

[25] Tang Q., Lu M., Xu B., Wang Y., Lu S., Yu Z., Jing X., Yuan J. (2021). Electroacupuncture regulates inguinal white adipose tissue browning by promoting sirtuin-1-dependent PPAR γ deacetylation and mitochondrial biogenesis.. Front. Endocrinol. (Lausanne).

[26] Li X., Hu Y., Wei C. (2022). To investigate the effect of Hexiaoyao powder on neuroinflammation in AD model rats based on Wnt/β-catenin signaling pathway.. Zhongguo Shiyan Fangjixue Zazhi.

[27] Li Y., Wang G., Li Y. (2024). Therapeutic potential of natural coumarins in autoimmune diseases with underlying mechanisms.. Front. Immunol..

[28] Szopa I.M., Majchrzak-Kuligowska K., Pingwara R., Kulka M., Taşdemir M., Gajewska M. (2025). A new method of canine CD4+ t lymphocyte differentiation towards the th17 phenotype with analysis of properties and mitochondrial activity.. Int. J. Mol. Sci..

[29] Albini A., Di Paola L., Mei G., Baci D., Fusco N., Corso G., Noonan D. (2025). Inflammation and cancer cell survival: TRAF2 as a key player.. Cell Death Dis..

[30] Yang X., Song N., Wang Z. (2019). Buzhong Yiqi Decoction can improve the immune disorder of mice with autoimmune thyroiditis by intervening miR-155 to regulate Th17 cells.. Zhonghua Zhongyiyao Xuekan.

[31] Cha Y., Liu J., Zhang W. (2024). To explore the mechanism of Buzhong Yiqi Decoction in treating chronic heart failure based on network pharmacology and molecular docking technology.. J. Univ. of South China.

